# Accelerating the Improvement of Human Well-Being in China through Economic Growth and Policy Adjustment

**DOI:** 10.3390/ijerph191912566

**Published:** 2022-10-01

**Authors:** Luhua Wu, Shijie Wang, Xiaoyong Bai, Guangjie Luo, Jinfeng Wang, Fei Chen, Chaojun Li, Chen Ran, Sirui Zhang

**Affiliations:** 1 School of Economics and Management, Tongren University, Tongren 554300, China; 2 State Key Laboratory of Environmental Geochemistry, Institute of Geochemistry, Chinese Academy of Sciences, Guiyang 550081, China; 3 Puding Karst Ecosystem Observation and Research Station, Chinese Academy of Sciences, Puding 562100, China; 4 Guizhou Provincial Key Laboratory of Geographic State Monitoring of Watershed, Guizhou Education University, Guiyang 550018, China; 5 School of Economics and Management, Liupanshui Normal University, Liupanshui 553004, China

**Keywords:** GPI, economic growth, human well-being, ecosystem services, China

## Abstract

Human well-being in many countries lags behind the gross domestic product (GDP) due to the rapid changes in the socio-economic environment that have occurred for decades. However, the mechanisms behind this complex phenomenon are still unclear. This study revealed the changes in human well-being in China from 1995 to 2017 by revising the genuine progress indicator (GPI) at the national level and further quantified the contribution of interfering factors that have driven the increase in the GPI. The results indicated that: (1) The per capita GPI of China showed an increasing trend with an annual growth rate of 12.43%. The changes in the GPI followed the same pattern as economic development, rather than presenting the phenomenon of economic growth combined with a decline in welfare that has been recorded in some countries and regions. (2) The increase in human well-being was mainly driven by economic growth, but it was most sensitive to social factors. (3) Increasing income inequality and the cost of lost leisure time contributed obvious negative impacts (24.69% and 23.35%, respectively) to the per capita GPI. However, the increase in personal consumption expenditures, the value of domestic labor, ecosystem service value, and net capital growth accelerated the rise in the GPI, with positive contribution rates of 30.69%, 23%, 20.54%, and 20.02%, respectively. (4) The continuous increase in economic investment and the strengthening of social management due to policy adjustments completely counteracted the negative impacts on human well-being, thus leading to a great increase in the per capita GPI. Such insights could provide theoretical support for decision making and policy implementation to improve global human well-being.

## 1. Introduction

The improvement of human well-being expands countries’ wealth, increases people’s incomes and consumption, and benefits various welfare levels. Therefore, human well-being has always been a focus of attention for all countries. So far, economic development has been considered by most countries as one of the best ways to improve human welfare. Almost all countries have made efforts to achieve a higher gross domestic product (GDP) through economic development to improve human welfare [1,2,3,4]. Although economic development has significantly improved human well-being over the decades, current research shows that the correlation between economic growth and human well-being in many countries is not as strong now as it was in the past 50 years [5,6,7,8,9,10]. Sustained economic growth does not automatically translate into higher welfare levels, as might be expected [11,12,13]. The idea that “a rising tide lifts all boats” is no longer suitable for describing the relationship between economic development and human welfare. On the contrary, long-term attention to GDP growth has led to the undesirable phenomenon whereby human well-being growth lags behind economic changes in many countries and regions [14,15,16,17]. Many countries, especially developed countries, have detected a clear “economic growth limit” or “relative threshold effect” in the process of improving human welfare [1] and have tried to adjust economic development and implement a series of livelihood policies to prevent or delay this phenomenon. However, many countries have formulated unreasonable economic policies in an attempt to promote human welfare due to the obscurity of the influence mechanisms between economic growth and human welfare evolution, such as blind expansion and planned economies. Unfortunately, these unreasonable economic policies have not only failed to improve human well-being, but have also led many countries to fall into the double predicament of economic stagnation and a decline in human welfare. Thus, quantifying the changes in human well-being at the national level will provide an important contribution to tracking global progress in this field and identifying priorities for decision making and policy implementation in relation to the achievement of sustainable development to enhance human well-being.

To address these challenges, scientists have made many efforts to evaluate human well-being. The genuine progress indicator (GPI) established by Cobb et al. in 1995 [18] based on the index of sustainable economic welfare (ISEW) was first applied to evaluate the relationship between economic development and social welfare in the United States. The abnormal phenomenon of GDP growth combined with welfare decline was revealed by the results of this evaluation. Subsequently, human well-being assessment quickly attracted a great deal of attention and research interest from most countries, including Canada, the United Kingdom, Australia, Italy, India, and Brazil [1,8,10,19,20,21,22,23,24]. These studies assessed the relationships between welfare changes and economic growth, resource consumption, and social management through the GPI, which was chosen as the official index to evaluate sustainable economic welfare in Finland, Australia, Maryland, and other regions. Currently, the GPI is widely recognized as an effective tool for evaluating the sustainable development level of a country or region [25,26,27,28] and providing a relatively feasible approximation of its economic welfare [8]. Moreover, previous studies have continuously improved the GPI system to conduct localized research and have tried to clarify the significant relationship between economic development and human well-being in their respective regions through comparative analyses of the changes in per capita GPI and GDP [15,24,29]. After decades of efforts, researchers have put forward the concept of development limitation [30,31,32] and the relative threshold effect to explain welfare decline [1,5], and it is widely believed that the positive social benefits of economic development are gradually offset by its huge negative environmental factors [2]. In the past, only a single timescale could be used for comparative studies, and the time-frequency evolution relationship between the factors could not be studied in depth from the perspective of multiple timescales. It is difficult not only to determine the key influencing factors in the relationship, but also to clearly define the contribution of economic growth to changes in human welfare, which has hindered our understanding of the relationship between various influencing factors and human welfare. As a result, many countries and regions are still unable to formulate scientific and reasonable social and economic policies to improve human well-being.

China, the most populous developing country, has experienced rapid economic development and various policy adjustments in the past few decades and has become the world’s second largest economy after the United States. However, China has also faced great socio-economic challenges (high crime and unemployment rates and income and gender inequality) and environmental challenges (natural disasters, climate change, air and soil pollution, water shortages and pollution, excessive resource consumption, low resource utilization, and energy shortages). Nevertheless, China is striving to improve its human well-being under the great pressure of limited resources, environmental issues, and socio-economic challenges. Understanding the variations in human well-being in China is significant and beneficial to its sustainable social development. Therefore, this study assumed that the evolution in human well-being has a mutual feedback relationship with economic development and social policy adjustment; this assumption determined our research objectives. The scientific goal of this study was to solve the following three problems: (1) How has the sustainable development of human well-being in China evolved at the national level? (2) What are the typical characteristics of the evolutionary relationship between human well-being and economic growth? (3) What are the key drivers of human well-being development and how have their contributions changed? 

To answer these questions, a revised GPI for China including 27 indicators was used to assess the changes in human well-being against the background of rapid economic growth and the gradual optimization of social development in China, relying on the annual national-level time-series datasets relevant to the GPI from 1995 to 2017. Then, the relative progress of and contributions toward different indicators were examined. We also detected the time-frequency variations in the GPI and GDP across multiple timescales using wavelet analysis. Finally, the Kaya identity was applied to decompose the total GPI, and the logarithmic mean Divisia index (LMDI) was used to quantify the relative contribution of demographic and economic effects to GPI change. This study could provide theoretical support for decision making and policy implementation to improve global human well-being.

## 2. Materials

The socio-economic dataset used to calculate the GPI in this study was obtained from different government departments in China and was compared and corrected through different data sources. In detail, the dataset was derived from the China Statistical Yearbook (http://www.stats.gov.cn/tjsj/ndsj/, accessed on 8 October 2019), China Financial Yearbook (http://www.mof.gov.cn/index.htm, accessed on 20 October 2019), China Environmental Statistics Yearbook (https://www.mee.gov.cn, accessed on 25 October 2019), China Education Statistics Yearbook (http://www.moe.gov.cn, accessed on 27 October 2019), China Health Statistics Yearbook (http://www.nhc.gov.cn, accessed on 28 October 2019), China Energy Yearbook (http://www.gov.cn/fuwu/bm/gjnyj/index.htm, accessed on 29 October 2019), China Population Statistic Yearbook (http://www.stats.gov.cn, accessed on 1 November 2019), China forestry statistical yearbook (http://www.forestry.gov.cn/, accessed on 2 November 2019), data compilation on time use in 2008 (http://www.stats.gov.cn/ztjc/ztsj/2008sjly/, accessed on 3 November 2019), and China Civil Affairs Statistical Yearbook (http://www.mca.gov.cn/article/sj/, accessed on 5 November 2019). The land-use data products involved in calculating ecosystem services values were obtained from the European Space Agency (http://maps.elie.ucl.ac.be/CCI/viewer/download.php, accessed on 8 March 2020), with a time span of 1995–2017 and a spatial resolution of 300 m. All indicators were converted to 1995 USD when calculating the GPI in order to eliminate the impact of inflation. For simplicity, US dollars is replaced by USD in this study.

## 3. Methods

### 3.1. Revision of GPI

To assess the real level of human well-being in China, we constructed a revised GPI considering China’s national conditions, which was composed of 27 indicators and included the three components of the economy, society, and the environment (Table 1). It not only took into account the possible damage to the ecological environment caused by economic development, but also considered people’s happiness beyond material satisfaction and could thus fully reflect the progress in human well-being. Referring to the GPI 2.0 and the other GPI systems used in different studies, we made some improvements to the three components of the model by adding several meaningful, reasonable, and monetized indicators; optimizing the calculation methods of certain indicators; and discarding other unreasonable indicators. 

First of all, we revised the economic component based on the Atkinson index. The personal consumption expenditures had to be adjusted according to income inequality in order to obtain weighted personal consumption expenditures, which were the basis of calculating the GPI. Thus, the GPI was finally obtained by adding or subtracting the remaining items. In previous studies, income inequality has mainly been measured by the Gini coefficient. However, China does not publish its Gini coefficient regularly, and its rationality for characterizing income inequality has been severely criticized. The Gini coefficient has no social welfare function and does not reflect the principle of reducing transfer. Additionally, it has long been criticized as a measure of income inequality that may reduce the per capita GPI by about 10% [17]. Therefore, we replaced the Gini coefficient with the Atkinson index. As a measure of welfare loss caused by income inequality, the Atkinson index, which was established to perform a social welfare function, highlights the social aversion to income inequality [33] and has been clearly recognized and applied in many countries and regions [12,34]. Based on their per capita disposable income, all families are divided into five equal groups (low income, lower middle income, middle income, upper middle income, and high income) by the China Statistics Bureau. Each group accounts for 20% of the total households. In recent years, the government statistics department has divided all residents into five groups. However, many years ago, residents were only divided into urban and rural residents. Therefore, the Atkinson index was first estimated for urban and rural residents, respectively, and the total Atkinson index by group weight was then calculated. In this study, the specific calculation formula of the Atkinson index followed Howarth and Kennedy [12] and Long and Ji [17].

Secondly, the environmental component was improved on the basis of considering the value of ecological services. Ecosystem services refer to the benefits that human beings obtain from the ecosystem. A large part of human well-being comes from ecosystem services, such as pollution purification, climate regulation, and soil and water conservation, which provide a basic guarantee for human survival. Additionally, the more services the ecosystem provides, the more is saved by people and the country on corresponding expenses, such as pollution control and water supply. However, the traditional GPI index system only considers the net change in the value of farmland, wetland, and forest, thus neglecting the various services, potential value, and sustainable benefits provided by other ecosystems [35], which may lead to the underestimation of the real level of human well-being [2]. Therefore, in the GPI 2.0 indicator system, we adopted the method of accounting for ecological service values proposed by Bagstad et al. [30] and took the results of Costanza et al. [36] as a reference to convert the ecosystem services provided by different land types into monetary values, which were included in the GPI index system as the main positive index of the environmental component.

The quality of the environment provided by the ecosystem plays an important role in promoting social stability, which can relieve negative psychological feelings and reduce the occurrence of various accidents. However, floods, earthquakes, other natural disasters, solid waste pollution, resource consumption, and CO_2_ emissions not only cause huge economic losses, but also may cause irreparable physical and mental injuries. In this study, we comprehensively estimated these indicators and further considered the welfare loss caused by environmental pollution and natural disasters in a more effective way. To account for the cost of water pollution, air pollution, solid waste pollution, noise pollution, and other forms of pollution, we referred to the total investment of the government in these issues.

Thirdly, the social component was also improved. Based on the research of Costanza et al. [25], we added an assessment of the non-defensive public expenditure on education and health, the defensive private expenditure on education and health, and services from public infrastructure according to the data availability of various management departments in China. These additional indicators could more reasonably reflect the real level of social welfare available to people while avoiding the calculation uncertainty of using other similar indicators.

All the revised indicators of the GPI could be quantified and calculated more accurately. See Table 2 for the specific calculation methods and processes.

### 3.2. Wavelet Analysis

The cross-wavelet transform (XWT) analysis was applied to detect the time-frequency characteristics of the GPI across multiple time scales and its potential influencing factors. Wavelet coherence (WTC) was calculated to analyze the periodicity correlation between the per capita GPI and its influencing factors. Wavelet analyses were performed using the free MATLAB R2017a software (Mathworks, Natick, MA, USA) with a code kindly written by Grinsted et al. [25] (http://noc.ac.uk/using-science/crosswavelet-wavelet-coherence, accessed on 16 October 2019). So far, wavelet analysis has also been widely used in the study of ecological hydrological processes to reveal the time-frequency evolution relationship of the two time series [40,41,42,43,44,45,46].

#### 3.2.1. XWT

Let us define two time series, *x_t_* and *y_t_*; their covariance is calculated by a cross-wavelet transform (XWT), which can be represented as:

(1) $$W_{t}^{XY}(z)=W_{t}^{X}W_{t}^{Y^{*}}(z)$$

where $W_{t}^{X}(z)$ is the wavelet transform of time series *x_t_* at frequency *z*, and $W_{t}^{Y^{*}}(z)$ is the complex conjugate of the wavelet transform for time series *y_t_*. The cross-wavelet spectrum can be calculated by:

(2) $$W_{t}^{XY}(z)=\left|W_{t}^{XY}(z)\right|e^{t\varphi_{t}(z)}$$

where $W_{t}^{XY}(z)$ is the power of the cross wavelet. The phase angle $\varphi_{t}(z)$ relates to the delay between the two time series at time *t* and frequency *z*, and can be denoted as:

(3) $$\varphi_{t}(s)=\tan^{-1}\left(\frac{I(Z(z^{-1}W_{t}^{XY}(z)))}{R(Z(z^{-1}W_{t}^{XY}(z)))}\right)\in \left[-\pi ,\;\pi \right]$$

where *Z* is a smoothing operator, and *I* and *R* are the imaginary and real parts of $W_{t}^{XY}(z)$, respectively.

#### 3.2.2. WTC

Wavelet coherence (WTC) was applied to study the coherence of the XWT in the time-frequency space and thus determine the intensity of the covariance of the two time series. The wavelet squared coherency $R_{t}^{2}(z)$ can be calculated as follows:

(4) $$R_{t}^{2}(z)=\frac{{\left|Z(z^{-1}W_{t}^{XY}(z))\right|}^{2}}{Z(z^{-1}{\left|W_{t}^{X}(z)\right|}^{2})\cdot Z(z^{-1}{\left|W_{t}^{Y}(z)\right|}^{2})}$$

where *Z* denotes a smoothing operator that can be written as:

(5) $$Z(W)=Z_{scale}(Z_{time}(W_{t}(z)))$$

where *Z_scale_* represents smoothing along the wavelet scale axis and *Z_time_* smoothing over time. For the Morlet wavelet, a suitable smoothing operator was provided by Torrence and Compo [47]:

(6) $${Z_{time}(W)|}_{z}={\left(W_{t}(z)\cdot c_{1}^{\frac{-t^{2}}{2z^{2}}}\right)|}_{z}$$

(7) $${Z_{time}(W)|}_{z}={\left(W_{n}(z)\cdot c_{2}\prod (0.6z)\right)|}_{n}$$

where *c*_1_ and *c*_2_ represent normalization constants, Π represents the rectangle function, and 0.6 is the empirically determined scale decorrelation length for the Morlet wavelet.

### 3.3. Kaya Identity

Demographic and economic growth were considered to be the main drivers of changes in human well-being. Thus, the Kaya identity was used to decompose the total GPI, and the driving factors of the GPI could be decomposed into two identity models:

(8) C=g×h

(9) C=p×k×e×f

where the Kaya identity reveals six driving factors of human well-being. Let *C* = GPI, which is the total welfare of the population; *g* = GDP; *p* = POP, which is the total population (POP), also called the population scale effect; *k* = GDP/POP, which is the per capita GDP, also called the economic scale effect; *e* = TPC/GDP, which is the total personal consumption (TPC) generated by the per unit GDP, also called the consumption intensity effect; *f* = GPI/TPC, which is the human well-being generated by the unit consumption level, also called the welfare structure effect; *h* = GPI/GDP, which is the human well-being generated by the per unit GDP, also called the economic welfare effect.

### 3.4. LMDI Decomposition Model

The additional decomposition of the LMDI was used to decompose the above Kaya identity of human well-being drivers. The total human well-being in the *t* period and the base period were defined as *C_t_* and *C*_0_, and the increment (Δ*C*) in human well-being from the base period to the *t* period could be expressed as

(10) $$\Delta C=C_{t}-C_{0}=\Delta C_{g}+\Delta C_{h}$$

(11) $$\Delta C=C_{t}-C_{0}=\Delta C_{p}+\Delta C_{k}+\Delta C_{e}+\Delta C_{f}$$

(12) $$D=\frac{C_{t}}{C_{0}}=D_{g}D_{h}D_{rsd}$$

(13) $$D=\frac{C_{t}}{C_{0}}=D_{p}D_{k}D_{e}D_{f}D_{rsd}$$

where Δ*C_g_* is the change in human well-being caused by the change in GDP; Δ*C_h_* is the change in human well-being caused by the change in economic welfare effect; Δ*C_p_* refers to the change in human well-being caused by the change in population scale effect; Δ*C_k_* refers to the change in human well-being caused by the change in economic scale effect; Δ*C_e_* refers to the change in human well-being caused by the change in consumption intensity; and Δ*C_f_* refers to the change in total human well-being caused by the change in welfare structure effect. The contribution rate of each effect is *D_g_*, *D_h_*, *D_p_*, *D_k_*, *D_e_*, and *D_f_*, respectively; *D_rsd_* = 1.

The calculation formula for the various effects is as follows:

(14) $$\Delta C_{i}=\frac{C_{t}-C_{0}}{\ln C_{t}-\ln C_{0}}\ln\left(\frac{i^{t}}{i^{0}}\right)$$

where *i* = {*g*, *h*, *p*, *k*, *e*, *f*}, Δ*C_i_* = {Δ*C_g_*, Δ*C_h_*, Δ*C_p_*, Δ*C_k_*, Δ*C_e_*, Δ*C_f_*}.

The calculation formula for the contribution rates of various effects after decomposition is as follows:

(15) $$D_{i}=\exp(\frac{\ln C_{t}-\ln C_{0}}{C_{t}-C_{0}}\times \Delta C_{i})$$

where *i* = {*g*, *h*, *p*, *k*, *e*, *f*}, *D_i_*= {*D_g_*, *D_h_*, *D_p_*, *D_k_*, *D_e_*, *D_f_*}.

## 4. Result Analysis

### 4.1. Changes in GPI and GDP

**Figure 1:**
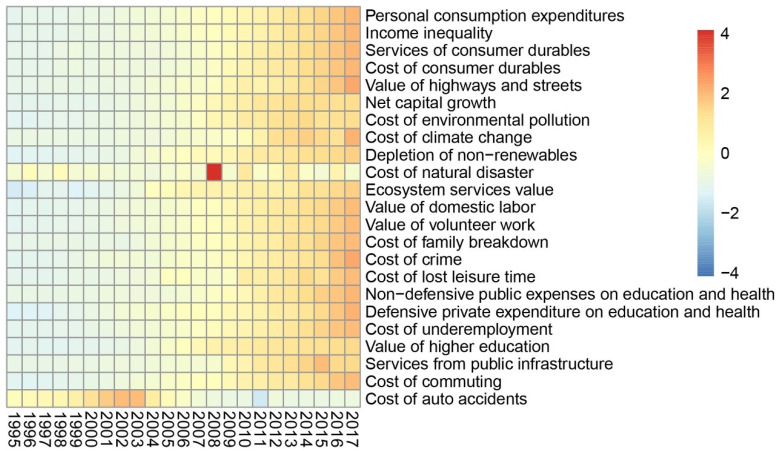
Change trends of standardized GPI indicators.

**Figure 2:**
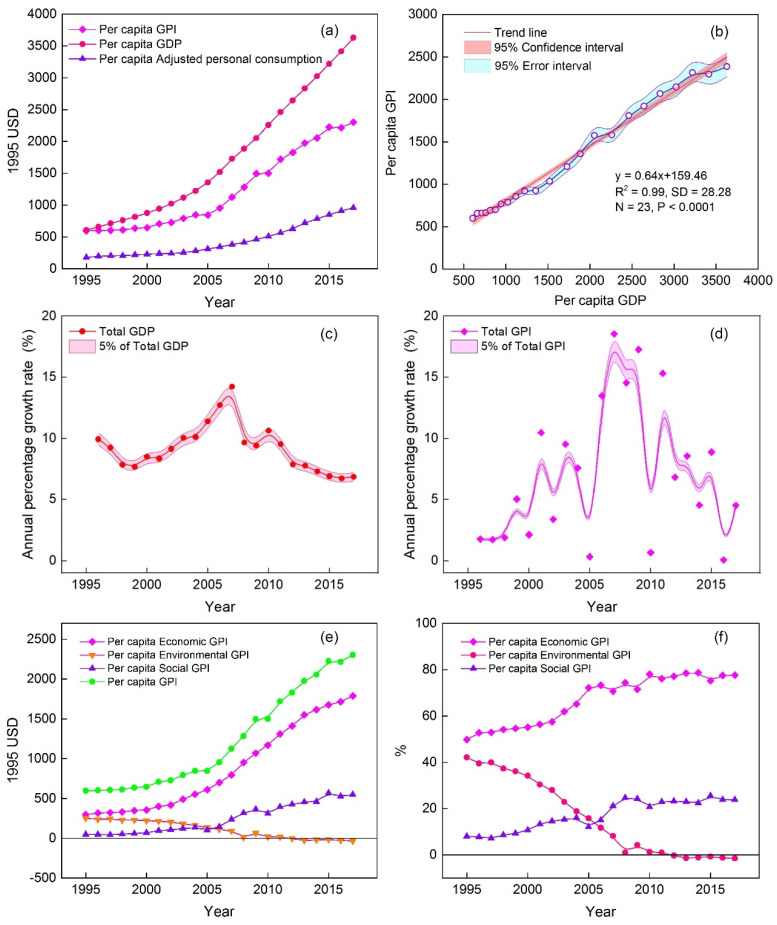
Characteristics of interannual evolution of GPI and GPI in China from 1995 to 2017: changes in per capita GPI and GDP (**a**); relative threshold effect detection (**b**); growth rate changes in total GPD and GPI (**c**,**d**); changes in per capita GPI for economic, environmental, and social components and their contributions to per capita GPI (**e**,**f**).

Since 1995, except for the reduction in the cost of auto accidents, the values (absolute values) of the indicators have increased, indicating that the negative impact caused by the increase in human well-being has also increased (Figure 1). As shown in Figure 2a, the per capita GPI and GDP have both risen, increasing by 2.86 and 4.98 times, respectively, with an average annual growth rate of 12.43% and 21.67%. However, the gap between the two has widened. Although the per capita GDP and adjusted per capita personal consumption expenditures are still soaring, per capita GPI growth slowed down significantly in 2016. The fact that the per capita GPI is lower than the per capita GDP indicates that the dividends of economic development have not been proportionally converted into human well-being. It is worth noting that the fluctuation range of the per capita GPI was significantly greater than that of the per capita GDP, indicating that the sustained and stable growth of the economy does not guarantee a steady rise in human well-being. On the contrary, the widening gap between the two shows not only that the GPI lags behind the GDP, but also that increased economic welfare may be offset by the negative impact of economic activities. From another point of view, these results suggest that the relative threshold effect may impact the relationship between economic development and human well-being, i.e., the sustained growth of the economy may lead to the alleviation of GPI growth, before the threshold of human well-being growth is reached, and, finally, it exhibits a downward trend. Although the relative threshold effect has been detected in many developed countries, China’s GPI has shown an upward trend since 1995 (Figure 2b). Although the upward trend slowed down in 2016, the GPI is still growing with economic growth, and the overall correlation coefficient between the two has reached 0.99. This insight was confirmed by the growth rate changes in the GDP and GPI. It was also found that the GPI showed an overall upward trend from 1995 to 2007 with increasing GDP, but then the growth rate of the GPI decreased with the decline in the GDP growth rate (Figure 2c,d). This indicated that the changes in human well-being have followed the same pattern as the changes in economic development in China, rather than presenting the phenomenon of economic growth accompanied by welfare decline that has been reported in some countries and regions around the world. Considering that the growth rate of the GPI was obviously greater than that of the GDP, it can be said that although economic development determines human well-being changes, there are still many other directly contributing factors.

In this study, we decomposed the total GPI into three components (economic, environmental, and social (Figure 2e)) and calculated their contribution rate trends (Figure 2f). The results showed that the growth rate of the economic GPI was slow before 2002, increased sharply after 2003, and slowed down until 2016. The growth rate of the social GPI increased obviously in 2006 but slowed down after 2014, while that of the environmental GPI decreased slowly, even changing from representing a positive effect to a negative effect after 2012, and finally remained flat with a stable contribution rate of about −1.5% after 2014. The economic GPI was the largest contributor to the total GPI, playing an important role in the sustainable growth of human well-being. Although the escalation effect was obvious before 2005, its contribution rate then increased slowly, and it maintained a relatively stable contribution of 77% after 2010. However, the contribution rate of the social GPI was smaller, with its highest rate of contribution being 25.53%, and it maintained a stable contribution rate of 24% after 2010. It can be concluded that the increase in human well-being was mainly driven by economic growth. Compared with the environmental GPI, which changed from offering a positive contribution to a negative contribution, the social GPI maintained a stable positive contribution.

### 4.2. Response of GPI to GDP on Annual Scale

**Figure 3:**
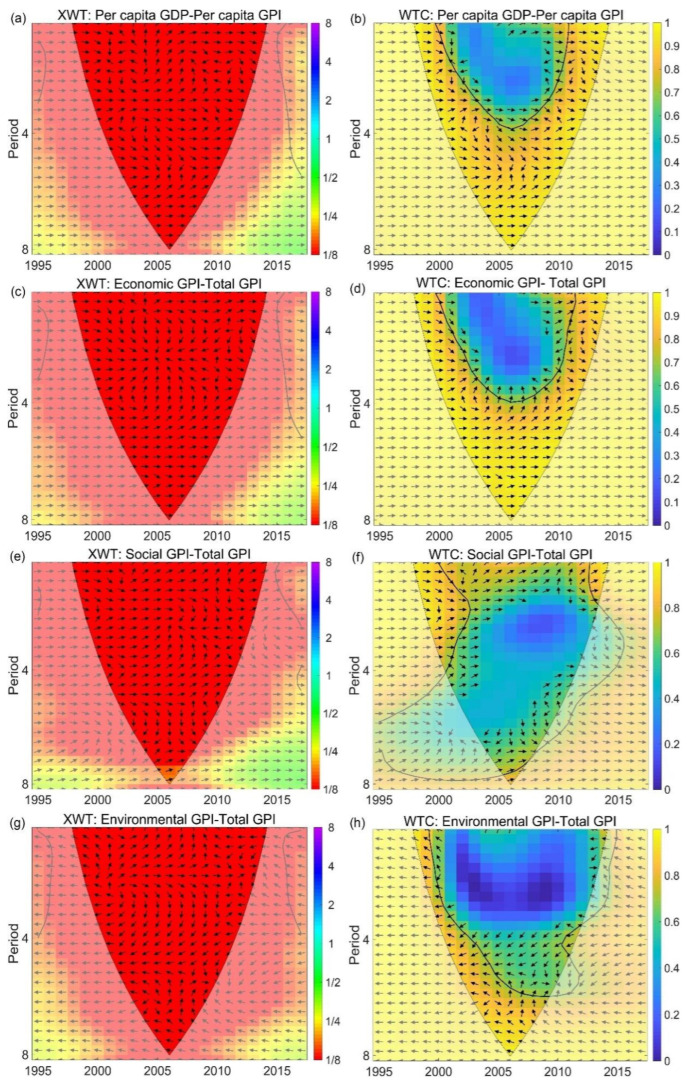
Wavelet power spectrum (**a**,**c**,**e**,**g**) and wavelet condensation spectrum of primary variables (per capita GPI and total GPI; and per capita GPI, economic GPI, social GPI, and environmental GPI) (**b**,**d**,**f**,**h**). The thick black contour designates the 5% significance level for red noise, and the cone of influence (COI) where edge effects might distort the picture is shown by a lighter shade. Phase change reflects the difference in response time of primary variables to influence factors. The phase relationship between influence factors and primary variables is indicated by arrows. The arrows from left to right indicate that the influencing factors and primary variables are in the same phase, which implies a positive correlation; the arrows from right to left indicate an inverse phase, which implies a negative correlation; the downward arrows indicate that influencing factors are 90° ahead of primary variables, and the upward arrows indicate that influence factors are 90° behind primary variables.

It was found that the gap between the per capita GPI and GDP expanded at different rates, with varying leading or lagging effects across different time scales. By using XWT and WTC, we analyzed the time-frequency relationship between the per capita GPI and GDP and further detected the lag times at different time scales. As shown in Figure 3a, the evolution relationship in the region that passed the red-noise standard spectrum test at the 0.05 significance level was very complex (the region surrounded by the thick black contour line), and the correlation was relatively low. However, a significantly high correlation was detected by WTC at the 4- and 5-year time scales. Three groups of significant regions showed obvious evolution characteristics between the per capita GPI and GDP, which were bounded by the 3- and 5-year time scales, respectively (Figure 3b). Significant regions in the 1–3-year time-scale bands were observed between 2000 and 2010, with a very complex evolution relationship wherein the positive and negative phases changed frequently. In these scale bands, it was also found that the per capita GPI synchronously increased with the per capita GDP before 1999, but was significantly ahead of the per capita GDP from 1999 to 2009, before gradually lagging behind. The lag effect was found in the significant regions of the 3–5-year time scales between 2000 and 2010, especially in the period between 2005 and 2008, where the phase angle range of 60–90° showed that the lag period was even longer than 2–3 years. A leading effect of 1–2 years was observed in the significant regions of the 5–8-year time scales between 2003 and 2010. For the regions that failed to pass the red-noise standard spectrum test at the 0.05 significance level, the evolution in the per capita GPI at each time scale before 2005 was consistent with that of the per capita GDP, before overtaking it at the 5–8-year time scale and lagging behind at the 3-year time scale. With the passage of years, the leading effect rapidly disappeared at the 5–8-year time scale, but the phase angle at the 3-year time scale gradually reduced from 45° to 30° after 2013, and the lag time finally stabilized at about 1.5 years.

By comparing the time-frequency evolution relationship between the total GPI and the per capita GPI for the economic, environmental, and social components, we found that the per capita GPI was more sensitive to the per capita social GPI, and the area of significant regions was 1–2 times greater than that for the other two components (Figure 3c,d). The distribution pattern of significant regions changed from a large time scale to a small time scale with the passage of time, and the time-frequency evolution became more complex (Figure 3e,f). The per capita economic GPI was mainly ahead of the total per capita GPI, while the per capita environmental GPI was negatively correlated with the total GPI (Figure 3g,h).

### 4.3. Contribution Proportions of Social, Economic, and Ecological Indicators to Increase in GPI

**Figure 4:**
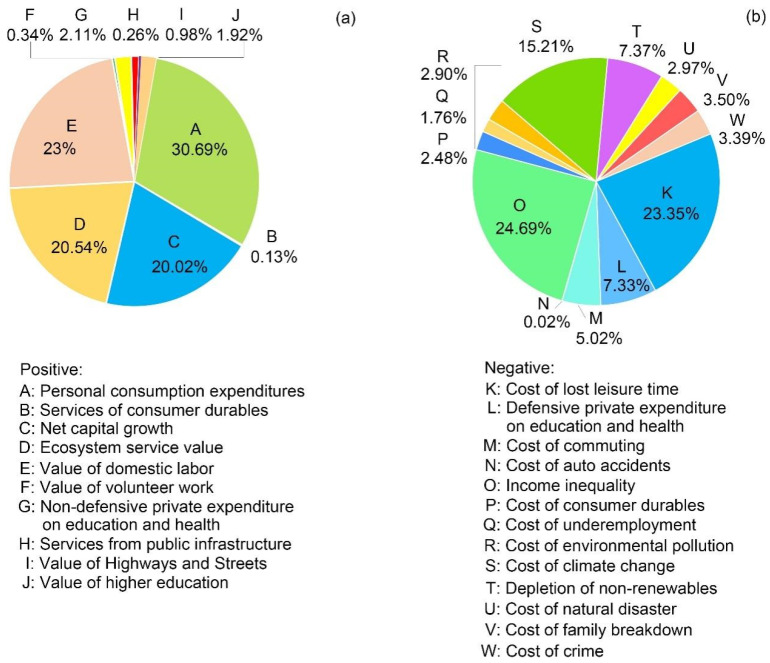
Average contribution of social, economic, and ecological indicators to total GPI from 1995 to 2017. (**a**) Average contribution of all positive indicators to total GPI; (**b**) Average contribution of all negative indicators to total GPI.

We further calculated the average contributions of all indicators to the total GPI and analyzed their change trends from 1995 to 2017. As shown in Figure 4a, personal consumption expenditures (30.69%), value of domestic labor (23%), ecosystem services value (20.54%), and net capital growth (20.02%) were the four major contributors to the growth of the GPI, accounting for 94.25% of the total contribution of positive indicators. As the starting point of GPI calculation, personal consumption expenditures increased by 55.33% from 39.29% in 1995 to 61.03% in 2017, and they now contribute the largest proportion (30.69%) to the total GPI of all positive-contribution indicators. From 1995 to 2017, the contribution of net capital growth to the total GPI increased by 72.84% from 21.28% in 1995 to 36.78% in 2017. The contribution of the value of domestic labor showed the fastest growth rate (132.11%) of all indicators, increasing from 21.02% in 1995 to 48.97% in 2017. Although the ecosystem services value showed an increasing trend, its contribution gradually decreased from 56.57% in 1995 to 13.61% in 2017, and the reduction rate has increased significantly since 2000.

Income inequality was the main factor hindering the rise in GPI (Figure 4b), accounting for 24.69% of the total contributions of all negative indicators. The cost of lost leisure time was also notable, accounting for 23.35%. Together, these factors accounted for 48.04% of the total contribution of negative indicators, which exceeded the total contribution of the remaining 11 negative indicators. Additionally, the depletion of non-renewables and defensive private expenditure on education and health were relatively prominent negative indicators, accounting for 15.21%, 7.37%, and 7.33% of the total negative GPI, respectively. The total contribution of the remaining indicators was relatively small, only accounting for 22.05% of the total negative contributions.

**Figure 5:**
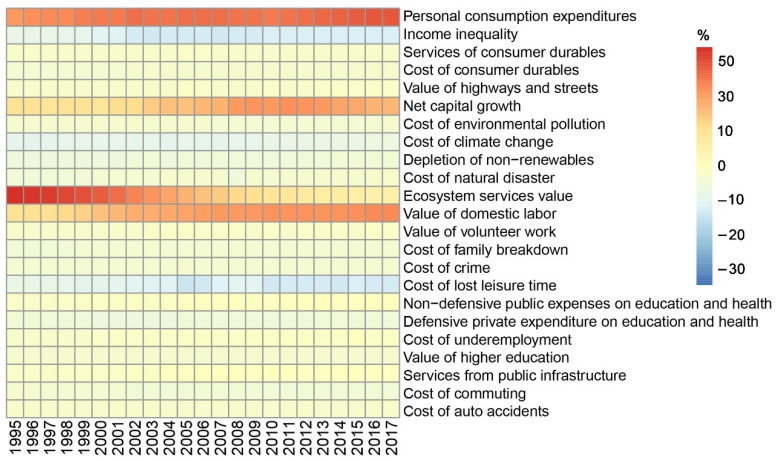
Contribution changes of social, economic, and ecological indicators to total GPI from 1995 to 2017.

The negative contribution rate of income inequality increased significantly (1.1-fold) from −9.27% in 1995 to −19.42% in 2017. The cost of lost leisure time showed the most dramatic negative contribution change, with an increase of 1.5 times from −8.29% in 1995 to −20.83%, and it is now the leading factor for reducing human well-being. The cost of climate change was the third main cause of reductions in human well-being, with less pronounced fluctuations and an average negative contribution rate to the total GPI as high as −10.56% (Figure 5). Most of the remaining 11 negative indicators contributed less than 5% to the total GPI, exhibiting less dramatic fluctuations.

In general, although the contribution of several negative indicators increased, the contribution rate of other negative indicators showed a slight downward trend, especially the cost of natural disasters, the cost of consumer durables, the cost of climate change, and the cost of auto accidents. The main positive indicators, in terms of both contribution value and contribution rate, all showed obvious growth, and most of the other positive indicators also showed a slight upward trend.

### 4.4. Relative Contribution of Demographic and Economic Effects to GPI Changes

**Figure 6:**
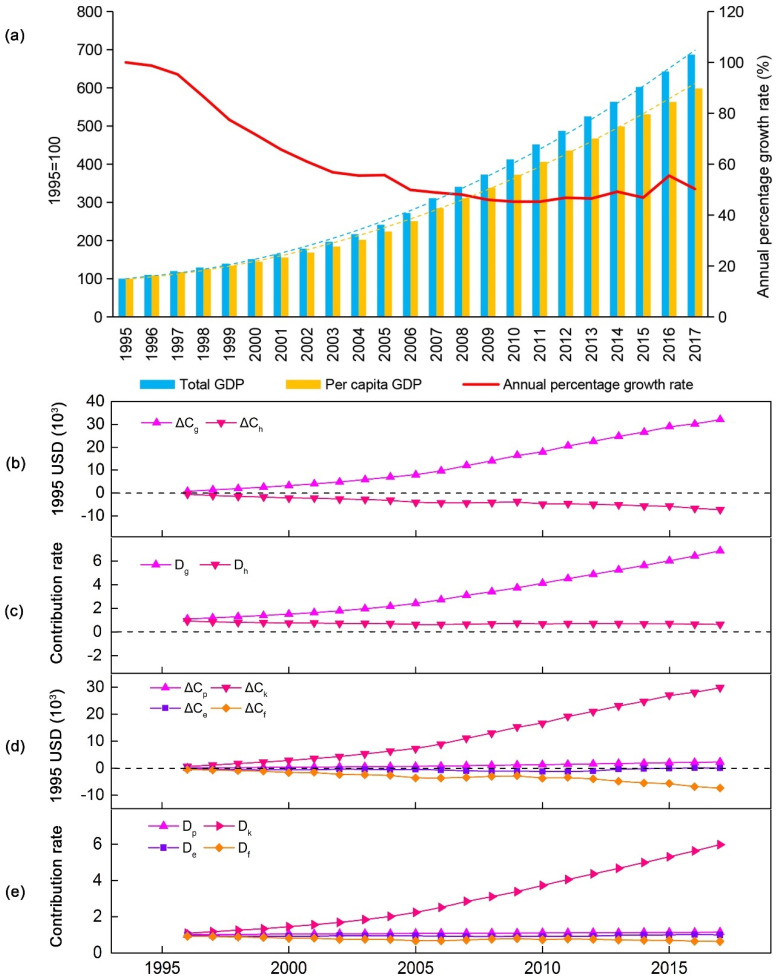
The contribution of different demographic and economy effects to the total GPI. (**a**) Indicators of real GDP, real per capita GDP, and population growth rate in China from 1995 to 2017. (**b**,**c**) represent the contribution value and contribution rate of g and h to total GPI, respectively. (**d**,**e**) represent the contribution values and contribution rates of *p*, *k*, *e*, and *f* to total GPI, respectively.

Since 1995, the total GDP and per capita GDP have maintained a rapid growth trend, increasing by 5.87 and 5 times, respectively. The growth rate of the population declined by a large margin and has then increased slowly. As a result, the total population has increased by 14.77%, resulting in an increasing gap between the growth rate of the per capita GDP and the total GDP (Figure 6a).

To reveal more clearly the influence of demographic and economic effects on human well-being, two Kaya identities were constructed and the LMDI model was used to decompose the GPI year by year, with a base year of 1995. Then, the relative contribution values and contribution rates of *g*, *h*, *p*, *k*, *e*, and *f* to the total GPI were estimated as follows. It was found that economic growth was the main driving factor for the improvement of human well-being, while population growth did not make a significant positive contribution to the improvement of human well-being (Figure 6b,c). During the period 1995–2017, the contribution value and contribution rate of the GDP to the GPI dramatically increased by 45.5 and 5.25 times, respectively, and they did not show a mitigation trend (Figure 6d,e). Similarly, the contribution value and contribution rate of *k* to the GPI also showed a sharp growth trend, increasing by 47.5 and 4.5 times, respectively. On the contrary, the contribution value of *p* to the GPI showed a very slow growth rate. Although the contribution value increased by 30 times, the contribution rate only increased by 13.86%. It is worth noting that the negative contribution values of *h* and *f* to the GPI increased by 12 and 13.6 times, respectively, and the negative contribution rates increased by 30% and 31%, respectively, which indicates that *h* and *f* were the main restraining factors of the growth of human well-being.

## 5. Discussion

### 5.1. Comparison with Previous Research Results

We concluded that the total GPI in China was on the rise from 1995 to 2017. This conclusion contradicts those of previous research in China based on GPI assessment using different indicators [1,8,17,38,48]. Previous results have concluded that the thresholds of GPI growth have been reached in China, as well as in many countries and regions worldwide [5,8,14,15,16,49,50,51,52]. This shows that sustained economic growth cannot increase the GPI indefinitely but will lead to the negative effect of welfare reduction. It has been reported that a per capita GDP of about USD 7000 (USD 2005) represents the limit of per capita GPI growth [8]. If this limit is exceeded, the GPI will be greatly reduced due to various social and environmental costs. However, the threshold effect was not detected in China in this study. Although the growth trend of the GPI is slowing down, we could conclude based on the Kaya identity and LMDI decomposition model that, so far, economic growth has played a positive role in promoting human well-being. Furthermore, although the threshold effect has been detected in some provinces of China, the total GPI in China is still on the rise, with no obvious threshold effect at play. This is because China has increased its net capital growth and non-defensive public expenses on education and health and has strengthened its social management while attaching importance to economic development, thus improving its human well-being. The per capita GPI changes were even ahead of the per capita GDP changes on the less-than-3-year time scale before 2010, according to our wavelet analysis. However, though the per capita GPI is lagging behind the per capita GDP, the lag time is shrinking significantly, which shows that China has promoted the improvement of human well-being in the economic and social dimensions through policy adjustment. Moreover, as shown in Table 3, the positive phenomenon of the common growth of the economy and human well-being has been observed not only in China, but also in Brazil, Japan, Poland, Greece, Italy, and other countries and regions where economic growth and social sustainability are coordinated and harmonious.

### 5.2. The Impacts of Economic Growth and Policy Adjustment on Human Well-Being

The contribution rates of the economic and social GPI to the total GPI have shown a stable trend. However, the human well-being loss caused by resource consumption and environmental pollution is increasing, which may be an important reason for human well-being decreases in the future. It is worth noting that natural disasters may also be an important factor in reducing people’s well-being. We clearly observed that the losses caused by natural disasters in 2008 were very different from those in other years. This was mainly because the 5‧12 Wenchuan earthquake of 8.0 magnitude occurred in the Sichuan Province of China on 12 May 2008. The seismic waves circled the earth six times, affecting more than half of China and many countries and regions in Asia. The damaged area totaled about 500,000 km^2^, causing 69,227 deaths, 17,923 instances of missing people, 374,643 injuries of varying degrees, and 19.93 million people to lose their homes, and the total affected population was 46.26 million. This resulted in a direct economic loss of CNY 845.14 billion and a natural disaster loss of CNY 1175.24 billion (USD 106.86 billion based on the USD exchange rate in 1995) in 2008, five times higher than that in 2007. In this study, we detected that the phenomenon of the GPI lagging behind the GDP was more prominent at the 4-year time scale, which may be a signal that economic growth poses a threat to the sustainability of natural resources and environments and social welfare. Although this may only be a misleading signal, China has taken several actions in various domains to avoid the ‘relative threshold effect’ of the GPI by changing its approach to development and making policy adjustments [59]. China has implemented a variety of policies to further eliminate poverty and to improve welfare, such as the medical reform, the confirmation of land rights, rural revitalization, and targeted poverty alleviation. Financial transfer investment rose from 29% in 1999 to 39.4% in 2010, marking an increase of 35.86% in the past decade. Driven by various development strategies and policies, China’s infrastructure construction and ecological protection have been greatly improved [60,61,62,63,64,65,66,67,68,69,70,71,72]. Since 1995, China’s economic investment has increased by 13.87 times in non-defensive public expenses on education and health, 35.02 times in the value of highways and streets, and 6.65 times in net capital growth. China has greatly reduced the effects of natural disasters by improving its ability to prevent them. By strengthening social management, the value of higher education and the value of domestic labor in China have increased by 8.5 and 9.32 times, and traffic accidents have been reduced by 50%. Additionally, we detected that the lag period of the GPI relative to the GDP after 2010 reduced significantly compared to previous years, and the lag has been maintained at 1.5 years since 2013, indicating that China has considered limiting blind expansion and highlighting the quality of its economic growth. It is undeniable that economic growth still inevitably accelerates investment in resource conservation, environmental pollution control, and emission reduction and aggravates certain social disharmony problems, such as violent crimes (imprisonment, homicide); traffic accidents; obesity; divorce; drug abuse; psychological depression; the loss of leisure time; and mental disorders, which affect human well-being and can hardly be eliminated in the short term. However, it is promising that the growth rates of the most negative indicators have shown declining trends since 2010. The growth rates of 9 out of 13 negative indicators show downward trends, which indicates that China has alleviated the growth of factors that can cause welfare loss through policy adjustment.

To continuously improve human well-being, first of all, China should continue to ensure steady, high-speed, and efficient economic development and should further eliminate poverty [73]. Secondly, China should improve its resource utilization efficiency, enhance environment protection, and realize the balanced development of resource consumption and economic growth, so as to reduce the welfare loss due to environmental issues. Finally, China should further strengthen the construction of a harmonious family atmosphere, improve work efficiency to reduce the loss of leisure time, further strengthen infrastructure and social management, and formulate more scientific and reasonable social public policies to achieve harmonious development between society and the environment.

### 5.3. Limitations and Future Research

In this study, we discarded certain indicators and added several new ones, mainly because the availability of data meant that some indicators lacked sufficient data, so these indicators were wither excluded or replaced by alternatives. The indicators established by different countries for evaluating human well-being are often partially replaced or adjusted according to the data availability, which leads to the disunity of the index system. Therefore, it is difficult to establish a GPI system that is applicable to all countries and regions. Because of the differences in our GPI index system and calculation methods, there may also be uncertainty in this study, and the results of the GPI evaluation may be different from those of previous studies.

At present, only the evolution relationship between people’s well-being and economic growth at the national level has been considered, thus ignoring the synergy and trade-off effects among different types of well-being. Future research should investigate further the trade-offs and synergy between different welfare goals, which will help reveal the complex mechanisms and consequences of human well-being. Achieving human well-being requires all relevant stakeholders to work together to reduce the barriers between different management and governance sectors as much as possible. Based on this, we could integrate effective methods to enhance collaborative well-being by identifying positive synergy and negative trade-offs between different welfare goals. Considering that economic development is closely related to human well-being and has the effect of globalization, future research should also be guided by the concept of whole-process coupling to study welfare changes, focusing on the spillover effect of actions in one region on the sustainable development of other regions in China, as well as the transnational spillover effect [74], or the impact of multiple surrounding and remote regions on the improvement of human well-being in the same region, not only limited to the efforts of local governments. Thus, many countries and regions may be able to achieve their welfare goals simultaneously by proposing, formulating, adjusting, and optimizing appropriate policies under the premise of steady economic development.

## 6. Conclusions

We revised certain indicators and calculation methods of China’s GPI, and the annual time series dataset related to the GPI at the national level from 1995 to 2017 was used to reveal the characteristics of the changes in the GPI according to wavelet analysis and to clarify the driving factors of human well-being according to the contribution rates of certain indicators, as determined by the Kaya identity and the LMDI decomposition model. The main conclusions were as follows:

(1) The per capita GPI of China showed an increasing trend with an annual growth rate of 12.43% at the national level from 1995 to 2017. Although the growth rate of the per capita GPI slowed down after 2016, it has not reached the growth threshold.
(2) The changes in the GPI have followed the same pattern as the changes in economic development in China, rather representing the phenomenon of economic growth accompanied by welfare decline that has been reported in some countries and regions.
(3) The contribution rates of most indicators promoting the growth of human well-being showed increasing trends, while the contribution rates of most indicators reducing human well-being declined after 2010, and the growth rates of 9 out of 13 negative indicators showed downward trends.
(4) The improvement of human well-being was mainly driven by economic growth, but it was most sensitive to social factors.
(5) The growth of personal consumption expenditures, the value of domestic labor, ecosystem services value, and net capital growth greatly improved human well-being, accounting for 94.25% of the total contribution of all positive indicators. Income inequality and the cost of leisure time loss were the two main factors that reduced human well-being, accounting for 48.04% of the total contribution of all negative indicators.

## Figures and Tables

**Table 1:** The indicators of the revised GPI.

Component	Item	Contribution	Classification
Economic component	Personal consumption expenditures	+	Built
	Income inequality	−	−
	Services of consumer durables	+	Built
	Cost of consumer durables	−	Built
	Value of highways and streets	+	Built
	Net capital growth	±	Built
Environmental component	Cost of water pollution	−	Natural
	Cost of air pollution	−	Natural
	Cost of noise pollution	−	Natural
	Cost of solid waste pollution	−	Natural
	Cost of other pollution	−	Natural
	Depletion of non-renewables	−	Natural
	Cost of climate change	−	Natural
	Cost of natural disasters	−	Natural
	Ecosystem service value	+	Natural
Social component	Value of domestic labor	+	Human
	Value of volunteer work	+	Human
	Cost of lost leisure time	−	Human
	Cost of commuting	−	Human
	Cost of family breakdown	−	Social
	Cost of crime	−	Social
	Non-defensive public expenses on education and health	+	Social
	Defensive private expenditure on education and health	+	Social
	Value of higher education	+	Social
	Cost of underemployment	−	Social
	Services from public infrastructure	+	Social
	Cost of auto accidents	−	Social

**Table 2:** Description of indicators and calculation processes of revised GPI.

Component	Idicators	Method	Data Source
Economic component	Personal consumption expenditures (+)	The starting point of GPI calculation based on the China Statistical Yearbook.	China Statistical Yearbook
	Income inequality (−)	Personal consumption expenditures × (1−Atkinson index). The specific calculation formula can be found in Long and Ji (2019) [17].	China Statistical Yearbook
	Services of consumer durables (+)	Durable goods stock × depreciation rate of 12.5%.	China Statistical Yearbook
	Cost of consumer durables (−)	Equals the sum of all household expenditure on consumer durables.	China Statistical Yearbook
	Value of highways and streets (+)	Total expenditures for streets and highways × 7.5% annual value [29].	China Statistical Yearbook
	Net capital growth (±)	Equals the difference between newly-added capital investment and the human capital required for such an increment. The specific calculation formula can be found in Long and Ji [17].	China Statistical Yearbook China Financial Yearbook China Labor Statistical Yearbook China Population and Employment Statistics Yearbook
Environmental component	Cost of water pollution (−)	The amount invested by the state in water pollution control.	China Statistical Yearbook China Environmental Statistics Yearbook
	Cost of solid waste pollution (−)	The amount invested by the state in solid waste pollution control.	China Statistical Yearbook China Environmental Statistics Yearbook
	Cost of air pollution (−)	The amount invested by the state in air pollution control.	China Statistical Yearbook China Environmental Statistics Yearbook
	Cost of noise pollution (−)	The amount invested by the state in noise pollution control.	China Statistical Yearbook China Environmental Statistics Yearbook
	Cost of other pollution (−)	The amount invested by the state in other pollution control.	China Statistical Yearbook China Environmental Statistics Yearbook
	Cost of climate change (−)	Social cost of carbon × total CO_2_ generated by fossil fuel combustion (USD/ton), USD 89.57/ton in 2000 [37].	China Statistical Yearbook China Energy Yearbook
	Depletion of non-renewables (−)	Fossil fuel consumption energy equivalent in oil barrels × substitution cost. The replacement costs of each non-renewable are: oil, USD 17.23/barrel; coal, USD 18.14/t; natural gas, USD 3.66/kCF (based on 1996 figures) [17].	China Statistical Yearbook China Energy Yearbook
	Cost of natural disasters (−)	Data were obtained from China Civil Affairs Bureau.	China Civil Affairs Statistical Yearbook
	Ecosystem service value (+)	The calculation was based on the value equivalent coefficient per unit area of each ecosystem provided by Costanza et al. [36].	European Space Agency China Forestry Statistical Yearbook
Social component	Value of domestic labor (+)	Hours spent on housework by gender × hourly wage for maids, housecleaners, and cleaners. This study only considered the population aged 15–64.	China Statistical Yearbook China Financial Yearbook Data compilation on time use in 2008
	Value of volunteer work (+)	Total hours of volunteer work × average opportunity cost (USD/h). This study only considered the population aged 15–64.	China Statistical Yearbook China Financial Yearbook
	Cost of lost leisure time (−)	Total hours of overtime × average opportunity cost (USD/hr)	China Statistical Yearbook China Population Statistic Yearbook Data compilation on time use in 2008
	Cost of commuting (−)	Total hours spent commuting × average opportunity cost (USD/h) + direct costs of vehicle purchase and maintenance.	China Statistical Yearbook China Financial Yearbook
	Cost of family breakdown (−)	Cost of divorce × number of divorces. The unit cost of divorce in China was USD 20427 in 2004 according to Costanza et al. [25] and Wen [38].	China Statistical Yearbook China Financial Yearbook
	Cost of crime (−)	Number of occurrances of each crime × victim cost estimate for each crime. Public security expenditure was substituted for crime cost in this study due to data unavailability.	China Statistical Yearbook
	Non-defensive public expenses on education and health (+)	Public expenses on education and health (i.e., the government paying for residents as a supplementary consumption expenditure of personal income) can improve welfare. Part of the public expenditure on health and education is defensive, so it does not promote public welfare and hence was excluded [39]. Referring to Pulselli et al. [19] and Bleys [11], non-defensive public education and health expenditure was defined as 50% of all public expenses on education and health.	China Statistical Yearbook China Health Statistics Yearbook
	Defensive private expenditure on education and health (−)	Part of the personal expenditure on education and health is defensive and was excluded from the personal consumption expenditures calculation. According to the research method of Long and Ji [17], the defensive private expenditure on education and health was defined as 50% of all private education and health expenditure.	China Statistical Yearbook
	Value of higher education (+)	Number of persons with a bachelor’s degree or higher education × social value of higher education [29].	China Education Statistics Yearbook
	Cost of underemployment (−)	Number of underemployed people × unprovided hours of constrained work × average hourly wage rate.	China Statistical Yearbook China Population and Employment Statistics Yearbook
	Services from public infrastructure (+)	Due to data limitations, the value of public infrastructure in this study was mainly based on the investment of the state in the field of transportation, which was similar to public education/health expenditure. It was not included in the personal consumption expenditures, but was considered.	China Statistical Yearbook
	Cost of auto accidents (−)	Number of crashes × average cost for injury or fatality (USD/incident). The cost and loss data of automobile accidents were provided by the national transportation department.	China Statistical Yearbook
GPI		Algebraic sum of all indicators based on their positive and negative contributions.	

**Table 3:** A summary of previous studies that have not found any sign of the threshold effect.

Region	Scope	Period	Study
US	Utah	1990–2007	Berik et al. (2011) [53]
	Chittenden, Vermont Burlington County	1950–2000	Costanza et al. (2004) [25]
	Northeast Ohio	1950–2005	Bagstad and Shammin (2012) [54]
	Hawaii	2000–2009	Ostergaard-Klem and Oleson (2013) [55]
	Fifty states	2011	Fox and Erickson (2018) [10]
Greece	National	2000–2012	Menegaki and Tsagarakis (2015) [23]
Italy	National	1960–1990	Guenno and Tiezzi (1998) [39]
	Siena	1999	Pulselli et al. (2006) [19]
	North, center, and south	1999–2009	Gigliarano et al. (2014) [22]
Brazil	National	1970–2010	Andrade and Garcia (2015) [24]
Poland	National	1980–1997	Gil and Sleszynski (2003) [56]
Japan	National	1970–2003	Makino (2008) [57]
	National	1970–2003	Kubiszewski et al. (2013) [8]
	National (rural and urban)	1975–2008	Hayashi (2015) [58]
China	National	1997–2016	Long and Ji (2019) [17]
China	National	1995–2017	This study

## Data Availability

The data analyzed in this study are subject to the following licenses/restrictions: The social and economic dataset can be accessed from the China Statistical Yearbook (http://www.stats.gov.cn/tjsj/ndsj/, accessed on 8 October 2019), China Financial Yearbook (http://www.mof.gov.cn/index.htm, accessed on 20 October 2019), China Environmental Statistics Yearbook (https://www.mee.gov.cn, accessed on 25 October 2019), China Education Statistics Yearbook (http://www.moe.gov.cn, accessed on 27 October 2019), China Health Statistics Yearbook (http://www.nhc.gov.cn, accessed on 28 October 2019), China Energy Yearbook (http://www.gov.cn/fuwu/bm/gjnyj/index.htm, accessed on 29 October 2019), China Population Statistic Yearbook (http://www.stats.gov.cn, accessed on 1 November 2019), China forestry statistical yearbook (http://www.forestry.gov.cn/, accessed on 2 November 2019), data compilation on time use in 2008 (http://www.stats.gov.cn/ztjc/ztsj/2008sjly/, accessed on 3 November 2019), and China Civil Affairs Statistical Yearbook (http://www.mca.gov.cn/article/sj/, accessed on 5 November 2019). The land-use data products involved in calculating ecosystem services values can be obtained from the European Space Agency (http://maps.elie.ucl.ac.be/CCI/viewer/download.php, accessed on 8 March 2020). Requests to access these datasets should be directed to jgywlh@gztrc.edu.cn.
